# Knowledge, Attitudes, and Practices Related to Sterilization Among Healthcare Workers at a Tertiary Care Hospital of Jharkhand: A Cross-Sectional Study

**DOI:** 10.7759/cureus.95275

**Published:** 2025-10-23

**Authors:** Surabhi Surabhi, Bhoopendra Singh

**Affiliations:** 1 Hospital Administration, Rajendra Institute of Medical Sciences, Ranchi, IND; 2 Forensic Medicine, Rajendra Institute of Medical Sciences, Ranchi, IND

**Keywords:** cssd, healthcare-associated infections (hais), hospital hygiene, infection control, knowledge attitudes and practices (kap), sterilization

## Abstract

Background: Healthcare-associated infections (HAIs) remain a major threat to patient safety in India, particularly in tertiary care teaching hospitals with high patient loads and limited resources. Effective sterilization of medical instruments is fundamental to infection prevention; however, healthcare workers’ (HCWs) knowledge, attitudes, and practices (KAP) directly influence its implementation.

Objective: The objective of this study is to assess the KAP regarding sterilization among HCWs at Rajendra Institute of Medical Sciences (RIMS), Ranchi.

Methods: A hospital-based cross-sectional study was conducted among 223 HCWs, including nurses, technicians, resident doctors, and support staff. Data were collected using a validated, semi-structured questionnaire evaluating demographics, professional characteristics, knowledge, attitudes, and practices related to sterilization. KAP scores were analyzed using descriptive statistics, ANOVA, and correlation tests.

Results: Of 223 participants, the majority were aged 31-40 years (45.3%), female (58.7%), and predominantly nurses (42.6%). Most worked in operating theaters (28.7%) and general wards (24.2%). Only 28.3% had formal infection control training, though 52.9% had attended sterilization-related training in the past two years.

The mean knowledge score was 14.2 ± 3.1 out of 20 (71.0%), with 23.8% scoring excellent, 41.7% good, 28.3% moderate, and 6.2% poor. Central Sterile Supply Department (CSSD) technicians scored the highest (16.8 ± 2.2) while support staff had the lowest scores (10.3 ± 3.6) (p < 0.001). Higher education and prior training were significantly associated with better knowledge levels (p < 0.001).

Attitudes were largely positive, with a mean score of 4.2 ± 0.6 out of 5; 78.5% demonstrated highly positive attitudes, particularly regarding patient safety (96.4%) and personal responsibility (94.2%). However, perceived barriers included insufficient resources (62.8%) and time constraints (58.7%).

Correlation analysis revealed significant moderate positive associations between knowledge and practice (r = 0.467, p < 0.001) and knowledge and attitude (r = 0.342, p < 0.001). Support staff demonstrated the weakest knowledge-practice relationship (r = 0.247, p = 0.012).

Conclusion: Sterilization knowledge among HCWs at RIMS, Ranchi was moderate overall, with clear disparities across professional categories and education levels. Attitudes toward sterilization were highly positive, but knowledge gaps, especially among support staff, and institutional barriers limited consistent practice. Regular, targeted training, competency evaluation, and improved institutional support are critical to strengthening sterilization practices and reducing HAIs in tertiary care hospitals.

## Introduction

Healthcare-associated infections (HAIs) continue to be a major challenge worldwide, posing serious risks to patient safety. These infections not only increase illness and healthcare costs but also contribute to the growing problem of antimicrobial resistance [1]. On any given day, about one in every 31 hospitalized patients is affected by at least one HAI [2]. According to the World Health Organization, out of over 421 million patients hospitalized globally each year, more than 42 million acquire these infections, highlighting the widespread impact on health systems everywhere [3]. Beyond their health consequences, HAIs also impose a hefty economic burden; for example, in the United States alone, they cost the healthcare system an estimated $96 to $147 billion annually [4]. These figures spotlight the critical need for better infection prevention and control measures to protect patients and reduce the strain on healthcare resources.

Effective sterilization of medical instruments and devices is a cornerstone of infection prevention and control, particularly in tertiary care hospitals where complex procedures and high patient loads are routine. Sterilization refers to the process of eliminating all forms of microbial life, including bacterial spores, and is measured by a sterility assurance level (SAL) of 10⁻⁶, which denotes a one in a million probability of a single viable microorganism surviving [5]. Commonly employed methods include steam sterilization, ethylene oxide gas, hydrogen peroxide plasma, and dry heat, selected according to the characteristics and intended use of the items being sterilized [6].

Despite advances in sterilization technologies and international guidelines, implementation remains vulnerable to human factors. Errors occurring during pre-cleaning, packaging, loading, or storage stages contribute significantly to sterilization failures [7]. Rutala and Weber note that lapses in sterilization processes often arise from human error, underscoring the critical importance of comprehensive training and strict adherence to standardized protocols to ensure effective sterilization outcomes. The knowledge, attitudes, and practices (KAP) of healthcare workers (HCWs) are thus fundamental to reducing sterilization failures [8,9].

In India, HAIs remain prevalent, with reported rates ranging from 4.4% to over 80% in different tertiary care settings, frequently linked to infrastructural gaps, inadequate training, and inconsistent adherence to sterilization standards [10].

The knowledge performance of staff was assessed through a semi-structured questionnaire, revealing that 50% of participants possessed complete knowledge, 20% had very good knowledge, 20% demonstrated good knowledge, and 10% had average knowledge. In evaluating practice performance, 90% of staff exhibited complete adherence to protocols, while 20% showed very good practice, 20% good practice, and 10% average practice. Notably, after the implementation of a targeted training program, awareness and competency improved significantly, with 99% of staff achieving higher scores post-intervention. These findings underscore the effectiveness of structured training programs in enhancing both knowledge and practical application among healthcare personnel [11].

In India, HAIs remain prevalent, with reported rates ranging from 4.4% to over 80% in different tertiary care settings-often linked to infrastructural gaps, inadequate training, and inconsistent adherence to standards. Wide variations persist in the design and functioning of Central Sterile Supply Departments (CSSDs), especially in semi-urban and resource-limited settings, undermining uniform infection prevention and control [12]. 

Given these persistent gaps, assessment of KAP related to sterilization among diverse HCW categories is essential to inform targeted interventions. This study was conducted at Rajendra Institute of Medical Sciences (RIMS), Ranchi, a tertiary care teaching hospital in Eastern India, aiming to identify competency deficits in sterilization procedures among HCWs and guide the development of context-appropriate training modules and institutional policies to strengthen infection control and reduce HAIs.

## Materials and methods

Study design

This study utilized a cross-sectional observational design to assess the KAP related to sterilization among healthcare workers (HCWs). Cross-sectional designs effectively capture perceptions and behaviors within defined populations at a specific time point, offering valuable institutional insights.

Study setting and population

The study was conducted at Rajendra Institute of Medical Sciences (RIMS), Ranchi, a tertiary care teaching hospital in Eastern India with over 2,200 inpatient beds and approximately 30,000-35,000 outpatient visits monthly. RIMS employs around 6,000 healthcare professionals, including doctors, nurses, technicians, and support staff.

Eligible participants included HCWs involved directly or indirectly in sterilization and infection control, such as CSSD staff, operation theatre nurses, resident doctors, surgical assistants, technicians, and support staff, with a minimum of three months’ experience at RIMS. Exclusions included administrative staff, individuals on extended leave, and those unwilling to participate. Data collection occurred over six months, from March to August 2025.

Sample size and sampling

Using standard cross-sectional formulas (Z = 1.96, P = 82.3%, d = 0.05), the minimum sample size was calculated as 223. Participants were recruited via convenience sampling, ensuring proportional representation across cadres and departments, facilitated by department heads.

Study tool and validation

Data were collected using a bilingual (English/Hindi), semi-structured questionnaire created by the authors containing four sections with a total of 26 items with a mix of open and closed items, after reviewing WHO infection prevention guidelines [13], CDC sterilization recommendations [14], and previously validated KAP instruments. The questionnaire is entirely original and self-developed; it contains four sections, in which the first section deals with demographic information. Out of the total seven items, one was open-ended and the remaining six were closed-ended. The second section dealt with knowledge of sterilization, containing seven closed-ended items. Attitude towards sterilization was assessed in the third section with a total of six closed-ended items. In the fourth section, practice related to sterilization was assessed using seven closed-ended items. It included sections on demographics, knowledge (multiple-choice questions), attitudes (Likert-scale items) [15], and practices related to sterilization. The questionnaire was assessed for content validity by a panel of subject experts who provided content relevance and content comprehensiveness, and representativeness for each item. Then, pilot testing of the questionnaire was conducted using a small number of participants to assess clarity, understandability, and feasibility of the items. Feedback from participant testing was then used to finalize the questionnaires used to collect data, and internal consistency was confirmed using Cronbach’s alpha ≥0.70 [16]. The complete KAP questionnaire used for data collection is provided in the Appendix (Tables 8-11).

Data collection and analysis

After obtaining ethical approval and informed consent, participants self-administered anonymized questionnaires on paper during non-peak hours with support from the study team. Data were entered into Microsoft Excel (Microsoft Corporation, Redmond, USA) and analyzed using IBM SPSS Statistics for Windows, Version 16 (Released 2007; IBM Corp., Armonk, New York, United States). Descriptive statistics summarized participant demographics and KAP. Associations between categorical variables were assessed using the chi-square (χ²) test, while comparisons of knowledge scores across professional and educational groups were performed using one-way ANOVA with Tukey’s HSD post-hoc test. Relationships among KAP scores were evaluated using Pearson’s correlation coefficient (r). Statistical significance was set at p < 0.05.

Ethical considerations

The Institutional Ethics Committee of RIMS approved the protocol (Approval No. 162) on 5th March 2025. Participation was voluntary with confidentiality and withdrawal rights assured.

## Results

Study population characteristics

A total of 223 HCWs participated. The majority were aged 31-40 years (45.3%), followed by 21-30 years (30.0%); 58.7% were female. Work experience varied, with 38.6% having 5-10 years, 28.7% with 1-5 years, and 20.2% with more than 10 years. In terms of professional category, nurses represented the largest group (42.6%), followed by technicians (23.8%), resident doctors (18.4%), and support staff (15.2%). Departmental distribution showed maximum participation from operation theatres (28.7%) and general wards (24.2%), followed by CSSD (18.4%), ICUs (16.1%), and emergency services (12.6%). Over half of HCWs worked day shifts (58.3%), while 28.7% worked rotating shifts and 13.0% in night shifts (Tables 1, 2).

**Table 1:** Demographic characteristics of participants (N=223) yrs: years; n: frequency; %: percentage Data are presented as frequencies and percentages. No inferential statistical tests were applied.

Characteristic	Category	n	%
Age group	21–30 yrs	67	30.0
	31–40 yrs	101	45.3
	41–50 yrs	42	18.8
	>50 yrs	13	5.8
Gender	Male	92	41.3
	Female	131	58.7
Experience	1–5 yrs	64	28.7
	5–10 yrs	86	38.6
	10–15 yrs	45	20.2
	>15 yrs	28	12.6

**Table 2:** Professional characteristics of participants (N=223) CSSD: Central Sterile Supply Department; ICU: Intensive Care Unit; n: frequency; %: percentage Data are presented as frequencies and percentages. No inferential statistical tests were applied.

Characteristic	Category	n	%
Professional role	Nurses	95	42.6
	Technicians	53	23.8
	Resident doctors	41	18.4
	Support staff	34	15.2
Department	Operating theatre	64	28.7
	General wards	54	24.2
	CSSD	41	18.4
	ICU	36	16.1
	Emergency	28	12.6
Shift pattern	Day shift	130	58.3
	Rotating shifts	64	28.7
	Night shift	29	13.0

Educational background

Of the participants, 41.7% held graduate degrees, 35.4% were diploma holders, 18.8% had postgraduate qualifications, and 4.0% had only certificate-level training. Specialized infection control training was reported by 28.3% overall, while 52.9% had attended sterilization-related training within the past two years (Table 3).

**Table 3:** Educational background and training (N=223) n: frequency; %: percentage Data are presented as frequencies and percentages. No inferential statistical tests were applied.

Category	n	%
Certificate course	9	4.0
Diploma	79	35.4
Graduate	93	41.7
Postgraduate	42	18.8
Infection control training	63	28.3
Training in past 2 years	118	52.9

Knowledge assessment

The mean overall knowledge score was 14.2 ± 3.1 out of 20 (71.0%), with a median of 15. Performance levels were classified as excellent in 23.8%, good in 41.7%, moderate in 28.3%, and poor in 6.2% (Table 4). Knowledge gaps were most pronounced in biological monitoring, quality assurance protocols, and newer sterilization technologies.

**Table 4:** Knowledge score distribution (N=223) SD: standard deviation; n: frequency; %: percentage Data are presented as frequencies, percentages, mean ± SD, median, and range. Differences in distribution across knowledge categories were analyzed using the chi-square (χ²) test. Statistical significance was set at p < 0.05.

Category	Score range	n	%
Excellent	17–20	53	23.8
Good	14–16	93	41.7
Moderate	11–13	63	28.3
Poor	<11	14	6.2

Knowledge by Professional Category

CSSD technicians achieved the highest mean scores (16.8 ± 2.2), followed by operating room nurses (15.9 ± 2.8). Resident doctors scored moderately (13.7 ± 3.4), while support staff recorded the lowest scores (10.3 ± 3.6). Differences across categories were statistically significant (p<0.001). Nurses performed stronger in theoretical aspects, while technicians excelled in practical applications.

Knowledge by Educational Level

Mean scores increased significantly with higher qualifications (p<0.001). Postgraduates had the highest mean scores (17.1 ± 2.0), followed by graduates (15.2 ± 2.6) and diploma holders (13.4 ± 3.1). Certificate holders had the lowest scores (9.8 ± 3.8). Infection control training was independently associated with better knowledge scores, irrespective of educational background (Table 5).

**Table 5:** Knowledge scores by professional category and education level CSSD: Central Sterile Supply Department; OR: Operating Room; SD: standard deviation; n: frequency; %: percentage Data are presented as mean ± SD, median, range, and percentage scores. Differences in knowledge scores across groups were analyzed using one-way ANOVA, with Tukey’s HSD test for post-hoc comparisons. Statistical significance was set at p < 0.05.

Category	n	Mean ± SD	Median	Range	%
CSSD technicians	41	16.8 ± 2.2	17.0	12–20	84.0
OR nurses	64	15.9 ± 2.8	16.0	10–20	79.5
Resident doctors	41	13.7 ± 3.4	14.0	7–19	68.5
General nurses	43	13.2 ± 2.9	13.0	8–18	66.0
Support staff	34	10.3 ± 3.6	10.0	5–17	51.5
Postgraduate	42	17.1 ± 2.0	17.0	13–20	85.5
Graduate	93	15.2 ± 2.6	15.0	9–20	76.0
Diploma	79	13.4 ± 3.1	13.0	7–19	67.0
Certificate	9	9.8 ± 3.8	10.0	5–15	49.0

Attitude assessment

The mean overall attitude score was 4.2 ± 0.6 out of 5 (84.0%), indicating predominantly positive attitudes. Most participants (78.5%) demonstrated highly positive attitudes (>4.0), while 18.4% had moderate-positive and 3.1% had neutral/negative attitudes. Nearly all participants (96.4%) acknowledged sterilization’s importance in preventing HAIs. Perceptions showed strong endorsement of patient safety (mean 4.7 ± 0.5) and personal responsibility (4.5 ± 0.6). However, adequacy of institutional support (3.8 ± 0.9), sufficient time for procedures (3.2 ± 1.1), and resource availability (3.4 ± 1.0) scored lower (Table 6). Minimal demographic variation existed: female HCWs scored slightly higher than male HCWs (4.3 ± 0.6 vs. 4.1 ± 0.7), and senior staff expressed stronger views on institutional responsibilities, though differences were not statistically significant.

**Table 6:** Attitude assessment results (N=223) HAI: healthcare-associated infection; SD: standard deviation; %: percentage Data are presented as mean ± SD and agreement percentages. No inferential statistical tests were applied.

Component	Mean ± SD	Agreement %
Patient safety as priority	4.7 ± 0.5	96.4
Personal responsibility	4.5 ± 0.6	94.2
Sterilization prevents HAIs	4.6 ± 0.6	95.1
Willingness for training	4.4 ± 0.7	89.7
Adequate institutional support	3.8 ± 0.9	71.3
Sufficient time	3.2 ± 1.1	58.7
Adequate resources	3.4 ± 1.0	62.8
Overall mean	4.2 ± 0.6	84.0

Correlation analysis

Significant associations were observed between knowledge, attitudes, and practices (Table 7). 

**Table 7:** Correlation analysis between knowledge, attitude, and practice K–P: Knowledge–Practice; r: correlation coefficient; p-value: probability value Relationships between knowledge, attitude, and practice scores were assessed using Pearson’s correlation coefficient (r). Statistical significance was set at p < 0.05.

Relationship	Correlation (r)	p-value	Interpretation
Knowledge–Attitude	0.342	<0.001	Moderate positive
Knowledge–Practice	0.467	<0.001	Moderate positive
Attitude–Practice	0.289	<0.001	Weak positive
Nurses (K–P)	0.428	<0.001	Moderate positive
Technicians (K–P)	0.521	<0.001	Strong positive
Support staff (K–P)	0.247	0.012	Weak positive

Knowledge correlated moderately with practice (r=0.467, p<0.001) and attitude (r=0.342, p<0.001), suggesting that improving knowledge may directly enhance practice adherence and indirectly foster positive attitudes. Attitude correlated weakly with practice (r=0.289, p<0.001).

By category, the knowledge-practice correlation was strongest among technicians (r=0.521), followed by nurses (r=0.428), and weakest among support staff (r=0.247, p=0.012). These findings highlight the pivotal role of structured training in translating knowledge into effective sterilization practices.

## Discussion

This study assessed KAP regarding sterilization among HCWs at a large tertiary care teaching hospital in Eastern India. The findings highlight an overall moderate level of knowledge, uniformly positive attitudes, but notable disparities across professional categories and educational levels.

Interpretation of findings

The mean knowledge score of 71% reflects moderate to good conceptual understanding, but with considerable variability. Only about one-fourth (23.8%) achieved excellent scores, while support staff recorded markedly lower mean scores (10.3 ± 3.6). These disparities mirror hierarchical differences in training exposure and responsibilities. Our data confirm that higher educational attainment and prior infection control training are significantly associated with better knowledge, consistent with prior reports from similar Indian tertiary hospitals [17,18].

Encouragingly, attitudes were overwhelmingly positive, with over 95% of participants emphasizing the role of sterilization in preventing HAIs. Strong endorsement of personal responsibility and willingness to attend training reflects staff readiness to comply with infection control practices if adequately supported. However, perceived barriers, including insufficient time and resources, underscore systemic constraints that may limit translation of positive attitudes into effective behavior [17,19].

Knowledge was moderately correlated with both attitude and practice, with the strongest knowledge-practice link observed among CSSD technicians. Most CSSD staff demonstrated adequate knowledge (57.4%) and good sterilization practices (55.6%), although positive attitudes were less common (24.1%). Knowledge showed significant positive correlations with both attitude (rs = 0.348, p = .010) and practice (rs = 0.302, p = .027). Nonetheless, gaps persisted in areas such as spill kit use and differentiation between scopes, underscoring the importance of continuous education and targeted training to enhance sterilization protocols and strengthen infection prevention and control [20].

Comparison with existing literature

Our findings align with previous KAP studies conducted in Indian [17], Saudi Arabian [20], and Nigerian [21] healthcare settings, which have consistently reported knowledge deficits among support staff and auxiliary workers, despite comparatively higher awareness levels among doctors and nurses. Similar trends observed across these regions suggest that disparities in knowledge and attitude are not confined to a single context but represent a broader challenge within healthcare systems. These findings highlight the importance of targeted, hands-on training interventions to bridge existing knowledge gaps and to cultivate more positive attitudes toward sterilization and infection control practices, particularly among CSSD personnel and support staff in diverse healthcare environments.

Similar studies from low- and middle-income countries report comparable gaps, particularly in understanding sterilization monitoring and quality control measures [22,23]. For example, studies in Nigeria demonstrated strong theoretical knowledge among nurses but limited consistency in practice due to infrastructural barriers [24].

Thus, while positive attitudes are ubiquitous, bridging the persistent knowledge-practice gap requires not only individual training but also robust institutional oversight-challenges particularly acute in resource-limited settings such as Eastern India.

Implications for policy and practice

The results of this study highlight several areas where changes in policy and practice could make a real difference. One of the most urgent needs is for targeted training programs that are not optional but required for all HCWs, especially support staff. A robust sterilization program, supported by adequate infrastructure, specialized equipment, and a skilled workforce, is essential for effective infection prevention. Quality assurance protocols in the CSSD, such as standardized sterilization procedures, regular monitoring with biological and chemical indicators, staff competency assessments, and systematic audits, ensure consistent adherence to these best practices. These programs align directly with the institution’s objective of minimizing hospital-acquired infections and ensuring patient safety by maintaining high standards of sterilization and reducing infection-related morbidity and mortality. The World Health Organization has long emphasized that structured, competency-based training is one of the most effective ways to improve infection prevention, particularly in settings where resources are limited and gaps in practice are most visible [25]. Alongside training, healthcare institutions would benefit from building routine competency assessments into their accreditation processes. By formally evaluating sterilization-related skills on a regular basis, institutions can improve accountability and ensure that staff maintain consistent standards of practice [26].

Another area that requires attention is resource allocation. Even the best training cannot succeed without the necessary tools. Facilities need to be equipped with reliable sterilization indicators, adequate instruments, and sufficient staffing. WHO guidance makes clear that ensuring access to essential supplies and human resources is fundamental to sustaining progress in infection prevention [25]. Finally, infection control should be seen not only as an individual responsibility but also as a system-wide priority. This means combining personal education with institutional supports such as standardized checklists, regular audits, and department-specific standard operating procedures. Together, these measures can help weave best practices into the daily routines of HCWs, making infection control part of the culture of patient safety rather than an additional task [25,26].

Strengths and limitations

The study’s strengths include its large and diverse sample from a high-volume teaching hospital, structured questionnaire development, and robust statistical analysis. However, the cross-sectional design limits causal inference, and reliance on self-reported practices may introduce social desirability bias. Additionally, convenience sampling, although stratified, may limit generalizability beyond comparable tertiary care institutions.

## Conclusions

This study highlights that HCWs at a tertiary care hospital in Eastern India generally exhibit positive attitudes toward sterilization and infection prevention, yet substantial gaps persist in knowledge and practice. CSSD technicians and nurses demonstrated higher competence, whereas support staff showed the greatest deficiencies. A strong association was observed between higher education, prior infection control training, and improved knowledge, underscoring the need for ongoing capacity-building initiatives. Strengthening sterilization practices requires targeted education for under-trained groups and enhanced institutional support, including sufficient resources, dedicated time, and systematic monitoring. Addressing both individual and organizational barriers is crucial to translating positive attitudes into consistent, effective practices.

## Appendices

**Table 8:** Questionnaire section A: demographic information

Question	Response Options
1. Participant ID	(Open response)
2. Age	a. 21–30 years b. 31–40 years c. 41–50 years d. >50 years
3. Gender	a. Male b. Female c. Other
4. Profession/Role	a. Doctor b. Nurse c. Technician d. Laboratory staff
5. Educational data	a. DM/MCh/Ph.D b. MD/MS/M.Sc c. MBBS/BDS/BHMS/B.Sc d. Diploma/GNM/Other
6. Years of experience in healthcare setting	a. 1–5 years b. 6–10 years c. 10–15 years d. >15 years

**Table 9:** Questionnaire section B: knowledge of sterilization

Question	Response Options
1. What does CSSD stand for in a hospital setting?	a. Central Surgical Services Department b. Central Sterile Services Department c. Central Supplies & Services Department d. Central Sterile Supplies Department
2. What is the primary purpose of sterilization in a healthcare setting?	a. To clean surfaces b. To destroy or eliminate all forms of microbial organisms c. To improve the aesthetic appearance of equipment d. To remove all types of dirt
3. Which types of sterilization methods are commonly used in your department?	a. Autoclaving (Steam Sterilization) b. Chemical Sterilization c. Dry heat d. Gas sterilization (ethylene oxide)
4. Do you know the correct time and temperature settings for autoclaving?	a. Yes b. No c. Somewhat d. Not sure
5. What is the minimum time required for autoclaving instruments at 121°C?	a. 5–10 minutes b. 15–20 minutes c. 30–35 minutes d. 60–65 minutes
6. Which of the following indicators is used to confirm sterilization in an autoclave?	a. Thermometer b. Biological indicator c. Chemical indicator only d. Pressure gauge
7. Which is the correct order of steps for reapplying surgical instruments?	a. Sterilization, Cleaning, Inspection, Packaging b. Cleaning, Inspection, Packaging, Sterilization c. Packaging, Cleaning, Sterilization, Inspection d. Inspection, Cleaning, Sterilization, Packaging

**Table 10:** Questionnaire section C: attitudes toward sterilization

Question	Response Options
1. How important do you think sterilization is in preventing infections in healthcare settings?	a. Very important b. Somewhat important c. Not very important d. Not important at all
2. Do you believe that sterilization protocols are well-followed in your hospital?	a. Yes b. No c. Sometimes d. Not sure
3. Do you think the hospital provides adequate training on sterilization techniques for staff?	a. Yes b. No c. Maybe d. Not sure
4. How confident are you in your own system to correctly sterilize medical equipment?	a. Very confident b. Confident c. Neutral d. Not confident
5. How important do you believe sterilization is in preventing hospital-acquired infections?	a. Very important b. Somewhat important c. Not very important d. Not important at all
6. Would you be willing to participate in further training or workshops on sterilization procedures and infection control?	a. Yes b. No c. Maybe d. Not sure

**Table 11:** Questionnaire section D: practices related to sterilization

Question	Response Options
1. How often do you follow sterilization protocols when handling medical equipment?	a. Always b. Often c. Sometimes d. Never
2. Which of the following does not kill endospores?	a. Autoclave b. Incineration c. Dry heat sterilization d. Pasteurization
3. Do you wear personal protective equipment (PPE) while performing sterilization tasks?	a. Always b. Often c. Sometimes d. Never
4. Do you follow the established sterilization protocol in your department?	a. Always b. Most of the time c. Occasionally d. Never
5. Do you document the sterilization process after each procedure?	a. Yes b. No c. Sometimes d. Not applicable
6. The most effective method for sterilizing surgical instruments in a hospital is?	a. UV radiation b. Boiling water c. Steam under pressure (autoclaving) d. Pasteurization
7. What steps do you take if you notice that sterilization equipment is malfunctioning?	a. Report to supervisor b. Try to fix it myself c. Wait for maintenance staff d. Ignore the issue
